# Inhaled nitric oxide for neonates with persistent pulmonary hypertension of the newborn in the CINRGI study: time to treatment response

**DOI:** 10.1186/s12887-018-1368-4

**Published:** 2019-01-12

**Authors:** Leif D. Nelin, Jim L. Potenziano

**Affiliations:** 1 The Research Institute at Nationwide Children’s Hospital, 575 Children’s Crossroads, Columbus, OH 43215 USA; 2 Mallinckrodt Pharmaceuticals, Bedminster, NJ USA

**Keywords:** Hypoxic respiratory failure, Inhaled nitric oxide, Oxygenation index, PaO_2_, Persistent pulmonary hypertension of the newborn

## Abstract

**Background:**

Substantial numbers of neonates with hypoxic respiratory failure (HRF) do not immediately respond to inhaled nitric oxide (iNO) and are often labeled as non-responders. This retrospective data analysis assessed time to treatment response in the iNO key registration trial.

**Methods:**

Treatment response was defined as a ≥10% increase in partial pressure of arterial oxygen (PaO_2_) or a ≥10% decrease in oxygenation index (OI) after initiation of study gas without the need for extracorporeal membrane oxygenation (ECMO). The proportion of patients showing a response at 30 min, 1 h, 24 h, and >24 h after iNO or placebo initiation was calculated and stratified by baseline PaO_2_ and OI.

**Results:**

Data from 248 patients (iNO: *n* = 126; placebo: *n* = 122) were included; 66 patients receiving iNO showed improvement in oxygenation without needing ECMO versus 38 receiving placebo. Of the 66 iNO responders, 73% responded within ≤30 min, 9% within ≤1 h, 12% within ≤24 h, and 6% after 24 h. Of the 38 patients with improvement in oxygenation without needing ECMO while receiving placebo, 53% showed improvement within ≤30 min, 16% within ≤1 h, 29% within ≤24 h, and 3% after 24 h. Baseline disease severity was not predictive of time to response. Of the 48 patients in the iNO treatment group who were classified as non-responders due to eventual need for ECMO and not included in the analysis of responders, 40 (83%) had an initial improvement in oxygenation during iNO therapy.

**Conclusions:**

Changes in PaO_2_ and OI after iNO initiation appear to be imprecise biomarkers of response to therapy in neonates with HRF. In some patients treated with iNO, it took up to 24 h to achieve improvement in oxygenation without need for ECMO, and a majority of those who eventually required ECMO did show an initial improvement in oxygenation during iNO treatment. Thus, reliable, objective, early criteria for iNO response still need to be established, and initial PaO_2_/OI responses should be interpreted with caution, particularly when considering discontinuing iNO therapy.

**Electronic supplementary material:**

The online version of this article (10.1186/s12887-018-1368-4) contains supplementary material, which is available to authorized users.

## Background

An estimated 2% of all live-born neonates require mechanical ventilation each year in the United States [1], and approximately 35,000 term and near-term neonates require mechanical ventilation because of hypoxic respiratory failure (HRF) [2]. Persistent pulmonary hypertension of the newborn (PPHN) is a common cause of respiratory failure in this population [3]. The risk of mortality in infants ≥34 weeks’ gestational age on mechanical ventilation is estimated at 5%; approximately 11% develop chronic lung disease and approximately 9% experience serious neurologic complications [3].

Inhaled nitric oxide (iNO), a potent, selective pulmonary vasodilator, is frequently used as adjunctive therapy in neonates with HRF associated with PPHN [3, 4]. The use of iNO has been shown to improve oxygenation [5–7], reduce the need for extracorporeal membrane oxygenation (ECMO) [5, 6], and reduce the risk of chronic lung disease [5] in newborns with PPHN and HRF. The Clinical Inhaled Nitric Oxide Research Group Investigation (CINRGI) study, the key registration trial of iNO in this patient population, showed that administration of low doses of iNO, initiated at 20 ppm and then titrated down to 5 ppm as tolerated, was associated with a significant reduction in the use of ECMO (38% in the iNO group vs. 64% in the placebo group; *p* = 0.001), a significant increase in the mean (SD) ratio of arterial-to-alveolar oxygen tension (0.10 [0.14] vs. 0.05 [0.13]; *p* = 0.02), and a significant reduction in the incidence of chronic lung disease, as defined by the need for supplemental oxygen at 30 days of age (7% vs. 20%; *p* = 0.02) [5]. These findings from the CINRGI trial demonstrated the effectiveness of iNO in this patient population and led to the approval of iNO by the US Food and Drug Administration (FDA) for these patients in 1999.

Although iNO has been shown to improve outcomes in this population, a substantial number of patients still do not have an immediate response to iNO and are often labeled as non-responders. A meta-analysis by Finer and Barrington found that iNO was clearly associated with improvement in oxygenation in infants with HRF [8]. However, the mortality rate for HRF in term and late preterm patients is approximately 11% and has remained relatively constant since the FDA approval of iNO in 1999 [9]. Therefore, interest is growing in developing rational approaches to treating term and near-term infants that include defining iNO responders so that other therapies, such as ECMO, can be initiated in a timely manner [4, 10–12]. Indeed, a lack of oxygenation response within 1 h has been suggested as a criterion for discontinuing iNO [4]. Furthermore, interest in other vasodilators for use in non-responders to iNO has been growing, although good evidence supporting their use is lacking [13–15].

The objective of the current analysis was to determine if there is a clear time to treatment response for term and near-term neonates using data from CINRGI, a placebo-controlled study [5]. We hypothesized that oxygenation responses within 1 h of starting iNO would not accurately predict the need for ECMO. A post hoc analysis of available data from the CINRGI study, which included patients treated with iNO or placebo, was performed.

## Methods

### Study population

Details of the design and methodology for the CINRGI study have been previously reported [5]. Briefly, the study included neonates with pulmonary hypertension who required assisted ventilation, had an oxygenation index (OI) ≥25, were born after 34 weeks’ gestation, and were ≤4 days old at the time of inclusion in the study. Neonates urgently requiring ECMO for refractory hypotension or profound hypoxemia were excluded from the study, as were those with a lethal congenital anomaly, substantial bleeding diathesis, active seizures, or history of severe asphyxia. Before randomization, patients were assigned to 1 of 5 pulmonary disease diagnostic categories: meconium aspiration syndrome, idiopathic pulmonary hypertension, pneumonia, respiratory distress syndrome, and lung hypoplasia syndrome. All physicians and nurses who provided care for neonates in the study were blinded to study treatments.

### Study design

The CINRGI study was approved by the institutional review board at each study site, and parents or guardians provided written informed consent. Patients were randomized to treatment with either iNO diluted in nitrogen (INO Therapeutics, Port Allen, LA) or nitrogen gas (Ohmeda, BOC Gases, Murray Hill, NJ) administered via an iNO delivery system into the inspiratory flow of the ventilator circuit. The placebo group received nitrogen. Study gas in both study groups was initiated at 20 ppm and continued for 4 h. After 4 h, the dose was decreased to 5 ppm if the patient’s condition was stable, partial pressure of arterial oxygen (PaO_2_) was ≥60 mmHg, and pH was ≤7.55. When response criteria were not met, study gas administration continued at 20 ppm and patients were re-evaluated every 4 h until the criteria were met or they received 24 h of study gas. During the first 24 h, if the PaO_2_ dropped to <60 mmHg when the fraction of inspired oxygen was 1.0, patients could be returned to the 20 ppm dose. After the first 24 h, patients experienced a dose decrease to 5 ppm; this dose was continued until patients achieved a fraction of inspired oxygen of <0.7, received 96 h of study gas, or were 7 days old, whichever came first. Treatment was considered a failure when patients could not tolerate the reduced dose at 24 h or when the study gas could not be discontinued within 96 h. The study gas was discontinued when patients were successfully weaned from it, when they met the failure criteria, or when they required ECMO. In cases requiring ECMO, the study gas continued until ECMO was initiated.

### Assessments

The primary outcome measure assessed in the main study was the use of ECMO in neonates treated with iNO compared with those not treated with iNO [5]. Other key outcomes assessed included improvement in the ratio of arterial partial pressure of oxygen-to-alveolar partial pressure of oxygen, incidence of short- and long-term complications, and death [5]. Although PaO_2_ and OI data were collected, the relationship between time on iNO and oxygenation responses was not examined in the CINRGI study.

### Statistical analysis

Descriptive statistics for baseline variables were calculated, and the distributions of values between the 2 study groups were tested using a *t*-test for continuous values and a chi-square test for categorical values. The main outcome of interest for the current post hoc analysis was the time from initiation of the study gas to achievement of treatment response, defined as a ≥10% increase in PaO_2_ or a ≥10% decrease in OI after initiation of the study treatment without the need for ECMO (standard criteria used to define treatment response at the time of the original CINRGI study). The proportion of patients achieving a successful response at 30 min, 1 h, 24 h, and >24 h after initiation of the study gas was calculated. Response rates at each time point were also stratified by baseline PaO_2_ and OI values.

## Results

A total of 248 neonates were enrolled in the original study; 126 patients were assigned to treatment with iNO and 122 were assigned to placebo [5]. The median duration of iNO treatment in the original study was 44 h [5]. Of the 248 neonates enrolled, 66 (52%) receiving iNO and 38 (31%) receiving placebo showed improvement in oxygenation and no need for ECMO. Baseline characteristics of responders in each study group are summarized in Table 1. The placebo group had a higher proportion of male patients than did the iNO group. The baseline mean PaO_2_ value tended to be higher and the baseline mean OI value tended to be lower in the iNO group than the placebo group; however, these differences were not statistically significant.

**Table 1:** Baseline demographic and clinical characteristics in patients showing improvement in oxygenation without needing extracorporeal membrane oxygenation

Variable	iNO group *n* = 66	Placebo group *n* = 38	*P* value
Birth weight, kg			
Mean (SD)	3.3 (0.55)	3.3 (0.65)	0.902
Age at enrollment, hours			
Mean (SD)	32.1 (20.63)	30.8 (18.25)	0.748
Male, *n* (%)	28 (42.4)	24 (63.2)	0.042
Race or ethnic group, *n* (%)			0.955
Black	28 (42.4)	14 (36.8)	
Hispanic	6 (9.1)	4 (10.5)	
White	29 (43.9)	18 (47.4)	
Other	3 (4.6)	2 (5.3)	
Primary pulmonary disease diagnosis, *n* (%)			
Meconium aspiration syndrome	23 (34.8)	14 (36.8)	
Idiopathic pulmonary hypertension	21 (31.8)	15 (39.5)	
Pneumonia	15 (22.7)	7 (18.4)	0.794
Respiratory distress syndrome	5 (7.6)	1 (2.6)	
Lung hypoplasia syndromes	2 (3.0)	1 (2.6)	
PaO_2_, mmHg			
Mean (SD)	87.8 (73.63)	66.2 (41.35)	0.058
Oxygenation index			
Mean (SD)	28.5^a^ (15.86)	33.2 (15.5)	0.154

*iNO* inhaled nitric oxide, *PaO*_*2*_ partial pressure of arterial oxygen

^a^The oxygenation index data are missing for 4 patients in the nitric oxide group

**Fig. 1:**
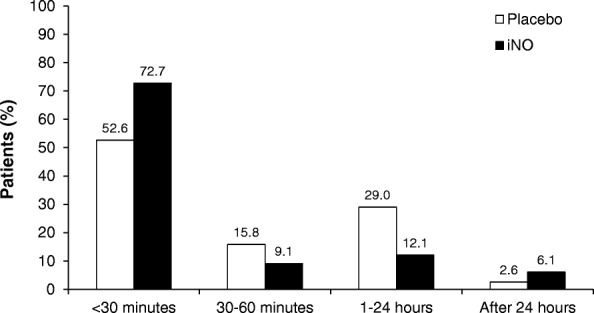
Time to improvement in oxygenation without need for extracorporeal membrane oxygenation among patients who showed response to treatment. *iNO*: inhaled nitric oxide

**Fig. 2:**
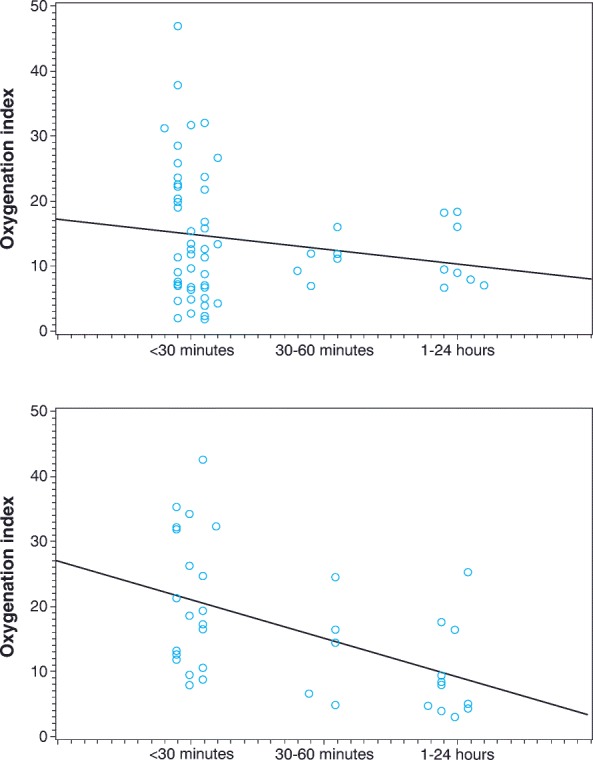
**a** Patients in the iNO group with improvement in oxygenation without needing ECMO stratified by baseline oxygenation index and time to improvement. **b** Patients in the placebo group with improvement in oxygenation without needing ECMO stratified by baseline oxygenation index and time to improvement

Of the 66 patients receiving iNO who showed improvement in oxygenation without needing ECMO, 48 (73%) responded within 30 min of treatment initiation, 6 (9%) within 1 h, 8 (12%) within 24 h, and 4 (6%) after 24 h (Fig. 1). Of the 38 patients receiving placebo who showed improvement in oxygenation without needing ECMO, 20 (53%) showed improvement within 30 min of treatment initiation, 6 (16%) within 1 h, 11 (29%) within 24 h, and 1 (3%) after 24 h. An analysis of patients who showed improvement in oxygenation without needing ECMO while receiving iNO or placebo, stratified by baseline OI and time to improvement, showed that disease severity at baseline (as reflected by OI) was not predictive of time to improvement in oxygenation (Fig. 2a and b). An analysis of the patients who had improvement in oxygenation without needing ECMO (ie, responders), stratified by baseline PaO_2_, showed similar results (data not shown).

In the CINRGI study, 48 of the 126 (38%) iNO-treated patients eventually required ECMO and were therefore labeled as non-responders. Forty (83%) of these patients eventually requiring ECMO had an initial response to iNO in terms of oxygenation, with all 40 having a ≥10% increase in PaO_2_ and/or a ≥10% decrease in OI, signifying an initial oxygenation response to iNO treatment. Interestingly, this group demonstrated a pattern of oxygenation response to iNO treatment similar to that of the iNO responder population described above, despite going on to need ECMO. Of the 40 patients who failed the primary outcome measure but had an initial response to iNO treatment, 22 (55%) initially responded to iNO within 30 min of treatment initiation, 7 (18%) within 1 h, 1 (3%) within 24 h, and 10 (25%) after 24 h. The mean (SD) PaO_2_ at baseline in the 29 patients who showed improvement in oxygenation within 1 h but eventually required ECMO was significantly lower than that in patients who had an oxygenation response to iNO treatment and did not require ECMO (46 [22] mmHg vs. 88 [74] mmHg; *p* < 0.0001). The mean (SD) OI at baseline was significantly higher in the iNO group with an initial oxygenation response who then went on to require ECMO than in the iNO-treated responders (47.2 [31.0] vs. 28.5 [15.9]; *p* = 0.0009). Of these 29 patients, 7 (24.1%) received ECMO within 4 h of initiation of iNO treatment, and 14 (48.3%) received ECMO within 12 h of initiation of iNO treatment.

## Discussion

Data from the CINRGI study clearly show a positive effect of iNO treatment on improving outcomes in term and late preterm patients with PPHN and HRF [5]. The CINRGI study was 1 of 2 trials that led to FDA approval of iNO for term and late preterm neonates with HRF for significantly reducing the need for ECMO therapy [5]. The studies used in obtaining FDA approval, including the CINRGI study, had very strict oxygenation eligibility criteria. Given the acuity of these patients, many units have, over time, lowered the oxygenation criteria for treating term and late preterm HRF patients with iNO, resulting in an increase in the use of iNO for patients with HRF without a marked change in mortality [9]. Meta-analyses have suggested that only approximately 50% of patients respond to iNO with an increase in oxygenation [8]. We undertook this post hoc analysis of data from the CINRGI study to determine if the physiologic response to iNO treatment was associated with time after treatment initiation. While many term and late preterm patients with HRF who responded to iNO treatment did so within 60 min, we found that there were patients who continued to exhibit an oxygenation response after 60 min (late responders), although no statistical difference was observed in the proportion of patients showing a late response in the iNO group versus the placebo group. This finding is consistent with the American Association of Respiratory Care published practice guidelines [4]. However, 60 min was not a clear, objective delineation of responders and non-responders; some patients in this study responded to treatment after 60 min, and some patients who showed improvement in oxygenation within 60 min of treatment initiation eventually required ECMO therapy. Thus, it seems that oxygenation responses in this population are complicated and can change relatively quickly. It is critical in term and late preterm neonates with HRF to continue monitoring their clinical status and to expect relatively rapid changes in oxygenation and clinical status. Furthermore, these data, taken together with the original outcome data from the CINRGI study, suggest that changes in oxygenation within 30 to 60 min of initiation of iNO treatment may not be a reasonable biomarker for whether the patient with HRF will ultimately avoid ECMO, the desired clinical outcome for iNO in PPHN. This finding may have important implications for the design of future randomized, controlled studies examining the use of iNO treatment to improve mortality in HRF.

When considering iNO treatment failure, it should be kept in mind that response to iNO treatment is influenced by the adequacy of ventilation and the level of lung recruitment. Kinsella et al. found that the combination of high-frequency oscillatory ventilation (HFOV) and iNO was more successful than either therapy given alone to neonates with HRF [16]. Similarly, Dobyns et al. found that, in pediatric patients with acute HRF, HFOV and iNO treatment improved oxygenation better than either therapy alone [17]. In both of these studies, the authors concluded that enhanced lung recruitment by HFOV enhanced the effects of iNO treatment [16, 17]. Thus, it is imperative that measures have been undertaken to optimize ventilation and lung recruitment before initiating iNO treatment.

Of the 48 patients treated with iNO who required ECMO in the CINRGI study, 40 had an initial response in terms of improved oxygenation, and 29 had an initial response within 1 h. These patients were more ill at baseline, as evidenced by a lower PaO_2_ and a greater OI than the group of iNO patients who showed an initial improvement in oxygenation and did not go on to require ECMO. The findings here are consistent with the notion that earlier treatment with iNO (ie, before OI is >25, as was the criterion in the CINRGI study) may be beneficial in avoiding ECMO in patients with HRF. For example, a 2-center study comparing early versus late iNO treatment in infants ≥35 weeks’ gestation with HRF found that patients treated earlier with iNO were less likely to develop severe HRF (OI >40) [18]; Konduri et al. found that early treatment with iNO (OI 15–25) resulted in fewer patients developing severe HRF, although this approach did not decrease the risk of mortality or ECMO use compared with standard iNO treatment [19]. In a European study of infants with a gestational age ≥33 weeks, early treatment with iNO was associated with a shorter length of mechanical ventilation and shorter length of stay in the neonatal intensive care unit [20]. Furthermore, early treatment with iNO has been found to be cost-effective by reducing the probability that patients will develop severe HRF [21, 22]. It is also reasonable to postulate that earlier initiation of iNO may affect the time to treatment response, and further studies may be needed to determine whether current use of iNO, which often is administered when OI values are lower than those needed to be eligible for the CINRGI study, have an effect on time to treatment response.

Potential limitations of this study should be considered. This retrospective analysis used data from a key clinical randomized, controlled trial of iNO in which the primary outcome measure was the need for ECMO. Our analysis assessed data reflecting the standard of care for treatment of neonates with pulmonary hypertension at the time the CINRGI study was conducted, which may limit interpretation of our results. Also, our analysis did not account for the potential impact associated with concomitant interventions and/or therapies that may have been used in the patients who did not show rapid improvement in oxygenation after initiation of study gas. Nevertheless, this is a unique data set from a relatively large group of patients with all of the necessary physiologic data to address our hypothesis. Some studies stopped iNO treatment in term and near-term patients with HRF after 30 to 60 min if there was no improvement in oxygenation. Thus, this data set from a study without stopping criteria for iNO treatment based on oxygenation response is necessary to address our hypothesis. All patients who were randomized to iNO in the CINRGI study received iNO treatment until their disease improved, they started ECMO therapy, or they reached 96 h.

This post hoc analysis of data from the CINRGI study suggests that while improvement in oxygenation tended to occur within 1 h of initiating iNO treatment in the majority of term and late preterm infants who responded to treatment, nearly 20% exhibited response after the first hour of iNO treatment. Also, an initial oxygenation response to iNO treatment within 1 h in this cohort did not necessarily mean that these patients avoided the need for ECMO.

## Conclusions

In summary, the results demonstrate that oxygenation response to iNO treatment in this patient population is complex and may change rapidly. Furthermore, the CINRGI study data are consistent with the notion that iNO treatment should be started early in the course of HRF for term and late preterm infants. The decision to stop iNO treatment may be complicated and should only be made after carefully considering all aspects of the patient’s condition, with particular attention on changes in mean airway pressure and fraction of inspired oxygen levels. These findings highlight the need for further clinical studies to develop a better understanding of factors associated with iNO non-responsiveness and how non-response to iNO affects outcomes to guide future efforts aimed at improving outcomes for term and late preterm patients with HRF.

## Additional file

Additional file 1: CINRGI Study Centers, IRB Names, and Lead Investigators. (DOCX 20 kb)

## Abbreviations

CINRGI: Clinical Inhaled Nitric Oxide Research Group Investigation
ECMO: Extracorporeal membrane oxygenation
FDA: Food and Drug Administration
HFOV: High-frequency oscillatory ventilation
HRF: Hypoxic renal failure
iNO: Inhaled nitric oxide
OI: Oxygenation index
PaO_2_: Partial pressure of arterial oxygen
PPHN: Persistent pulmonary hypertension of the newborn
