# Supplementary material for: Inhaled nitric oxide for neonates with persistent pulmonary hypertension of the newborn in the CINRGI study: time to treatment response
Source: BMC Pediatr. 2019 Jan 12;19:17. doi: 10.1186/s12887-018-1368-4 (PMC6330425; doi:10.1186/s12887-018-1368-4)
Supplement: Supplementary file 1 — CINRGI Study Centers, IRB Names, and Lead Investigators. (DOCX 20 kb) [file 12887_2018_1368_MOESM1_ESM.docx]

**Additional file 1**

**CINRGI Study Centers, IRB Names, and Lead Investigators**

| **Study Center** | **IRB Name** | **Lead Investigator** |
| --- | --- | --- |
| Egleston Children’s Hospital & Emory University  Atlanta, GA | Human Investigations Committee | Beverley J. Roy, MD |
| Grady Memorial Hospital  Atlanta, GA | Human Investigations Committee | L. Jain, MD  I. Seabrook, MD |
| Carolinas Medical Center  Charlotte, NC | Institutional Review Board | Thomas J. Kueser, MD |
| Greenville Memorial Hospital  Greenville, SC | Institutional Review Board | Whit Walker, MD |
| Richland Memorial Hospital  Columbia, SC | Office of Grants and Research Administration | David Marsh, MD |
| Arnold Palmer Hospital for Children & Women  Orlando, FL | Institutional Review Committee | Jose Perez, MD |
| Medical University of South Carolina  Charleston, SC | Institutional Review Board of Human Research | W. Michael Southgate, MD |
| Vanderbilt University Medical Center  Nashville, TN | Vanderbilt Institutional Review Board | William F. Walsh, MD |
| All Children’s Hospital  St. Petersburg, FL | Institutional Review Board | Anthony Napolitano, MD |
| Georgetown University Hospital  Washington, DC | Institutional Review Board  Research & Graduate Education | Martin Keszler, MD |
| University Hospital  San Antonio, TX | Office of Institutional Review Board  University of Texas Health Science Center at San Antonio | Michael Odom, MD |
| Christ Hospital and Medical Center  Oak Lawn, IL | Medical Investigation Committee | Monohar Rathi, MD |
| Ochsner Foundation Hospital  New Orleans, LA | Clinical Investigations Committee | Marie McGettigan, MD |
| University of South Dakota  Vermillion, SD | Institutional Review Board | Dennis Stevens, MD |
| Neonatology Associates  Phoenix, AZ | Institutional Review Board for Human Research | David Hall, MD |
| Duke University Medical Center  Durham, NC | Institutional Review Board | Reese H. Clark, MD |
| Children’s Hospital Medical Center  of Akron  Akron, OH | Investigational Review Board | Thomas Jeffrey Butler, MD |
| Wilford Hall Medical Center  Lackland AFB  San Antonio, TX | Committee for Investigational Research | Bradley A. Yoder, MD |
| Columbus Hospital  Chicago, IL | Institutional Review Board | Daksha Patel, MD |
| Crouse Hospital  Syracuse, NY | Institutional Review Board | Ellen Bifano, MD |
| Pennsylvania Hospital  Philadelphia, PA | Pennsylvania Hospital | Vinod Bhutani, MD |
